# The Real-World Utilization Pattern of Increased Utilization of Advanced Topical Adjunctive Hemostats in a Vertically Integrated Healthcare System

**DOI:** 10.36469/9813

**Published:** 2017-01-11

**Authors:** Lee Stern, Yi-Chien Lee, Biwen Tao, Lois Lamerato, Gaurav Gangoli, Richard Kocharian, Walter Danker III

**Affiliations:** 1 LASER Analytica, New York, NY; 2 Henry Ford Health System, Detroit, MI; 3 Ethicon, Inc., Somerville, NJ

**Keywords:** resource utilization, oxidized regenerated cellulose (orc), surgicel, surgery, bleeding, hemostat

## Abstract

Hemostasis products, such as SURGICEL®, have been increasingly used across a wide variety of surgical procedures to mitigate bleeding-related risks and complications. This retrospective observational study described the utilization pattern of the SURGICEL® family of oxidized regenerated cellulose products (SURGICEL® ORIGINAL, SURGICEL® FIBRILLAR™, SURGICEL SNoW®) in a large, vertically integrated healthcare system, by utilizing electronic medical records (EMR) extracted from August 2013 through June 2015 at Henry Ford Health System (HFHS). Descriptive measurements were compared between SURGICEL® ORIGINAL and advanced SURGICEL® products (SURGICEL® FIBRILLAR™ and SURGICEL SNoW®) for pooled common surgical procedures. Among 1471 patients, 450 received SURGICEL® ORIGINAL, and 1021 received advanced SURGICEL® products. A significantly greater proportion of patients given advanced SURGICEL® products had comorbidities (91.0% vs 85.6%, p=.0024), prior bleeding conditions (49.9% vs 30.9%, p<.0001), and prior use of anticoagulants (27.7% vs 5.3%, p<.0001). Advanced SURGICEL® products were more likely to be used in coronary artery bypass grafting (13.7% vs 1.6%, p<.0001). Among a sub-set of 1420 patients with complete package size information (988 Advanced and 432 ORIGINAL), significantly fewer mean normalized units of Advanced SURGICEL® were used per patient case (3.9 vs 5.5, p<.0001). Despite Advanced SURGICEL® products being utilized in higher risk bleeding situations compared to cases where SURGICEL® ORIGINAL was utilized, fewer overall normalized units of Advanced SURGICEL® were required per patient case. Further research is needed to investigate the implications of topical hemostat use in continuous oozing bleeding situations on outcomes, hospital costs, and resources.

## INTRODUCTION

All surgical procedures pose a risk of bleeding to the patient. Surgical site bleeding can cause complications of surgery and reoperation, and may lead to increased morbidity and mortality.[1,2] Uncontrolled bleeding is also associated with higher rate of transfusions, which can negatively affect health outcomes and increase the cost of healthcare.[3-5] Certain conditions may exacerbate bleeding, such as use of anticoagulant drugs and comorbidities including obesity, arterial hypertension, diabetes mellitus and renal dysfunction.[6,7] Among these factors, bleeding history and use of anticoagulants have been used as part of assessment tools to predict bleeding risk prior to surgical procedures.[7]

Topical hemostats are the most commonly used adjunctive hemostatic products[8], which have a mechanical surface to assist in clot formation. SURGICEL® topical hemostats consist of plant-derived oxidized regenerated cellulose (ORC). SURGICEL® ORIGINAL was the first SURGICEL® hemostat, a sheer woven ORC, and was approved by FDA in 1959. Advanced SURGICEL® products include SURGICEL® FIBRILLAR™, a fibrous ORC that can be peeled to form layers and tufts[9], and SURGICEL SNoW®, an ORC with non-woven structure to increase surface contact with the bleeding site. These two advanced products were approved in 1996 and 2012, respectively. Recent studies indicated that SURGICEL SNoW® and SURGICEL® FIBRILLAR™ have faster times to hemostasis compared to SURGICEL® Original.[10] Although both SURGICEL® Original and advanced SURGICEL® hemostat products have been introduced to control bleeding, little is known about how these products are being used by surgeons in clinical practice. Based on the superior hemostatic properties of advanced SURGICEL® products in comparison with SURGICEL® Original, we hypothesized that advanced SURGICEL® products would be more frequently used among patients with characteristics for higher bleeding risk, and fewer units of advanced SURGICEL® products would be used for each surgery compared to SURGICEL® Original.

This study aimed to describe the real-world utilization pattern of both SURGICEL® Original and advanced SURGICEL® products in the clinical practice of a vertically integrated healthcare system. The nature of a vertically integrated system allows for seamless capture of all aspects of patient care from the inpatient to the outpatient setting as well as details regarding the resources consumed within surgical procedures are captured within a comprehensive EMR system (Epic Systems Corporation). The patient characteristics related to bleeding risk and the units of product used for each surgery were compared between SURGICEL® Original and advanced SURGICEL® products for a diverse spectrum of pooled common surgical procedures.

## PATIENTS AND METHODS

### Study design

This was a retrospective observational study utilizing electronic medical records (EMR) extracted from the health care information software (Epic Systems Corporation).

### Data source

The data source for the study was the Henry Ford Health System (HFHS). The HFHS is a Michigan-based vertically integrated health system providing health insurance and healthcare delivery. In 2014, approximately 3.39 million outpatient visits and more than 73 000 surgical procedures were performed at the HFHS.[11] Surgery cases and other medical procedures were identified by ICD-9 and CPT codes. SURGICEL® usage records were extracted from surgery notes within Epic. Admission and discharge information were collected from patient encounter records.

## Study period

Data were extracted for any patients who had a hospital admission for a relevant surgery between August 6, 2013 and June 18, 2015 based on the availability of the EMR. The index date was the date of first surgery with SURGICEL® usage. The first surgery with SURGICEL® usage for each patient was defined as the index surgery.

## Study cohort

Only patients and surgery cases that met the following criteria were included in the analysis: 1) surgery was performed under inpatient setting; 2) patient aged ≥18 at index date; 3) received either a SURGICEL® Original or Advanced SURGICEL® product (patients who received both products were excluded from the analysis); 4) had identifiable and complete SURGICEL® usage records (with complete and verified information of product name for all SURGICEL® usage for the index surgery); 5) had related surgical procedures(as pre-specified in the analysis plan). Patients who had surgical procedures in more than one category were classified into a separate group as multiple surgeries.

## Descriptive measurements

Both categorical and continuous variables were assessed for patient demographics and characteristics, surgery types, SURGICEL® usage, and health outcomes. Since both SURGICEL® Original and advanced SURGICEL® products are available in a series of package sizes, the units of SURGICEL® used per surgery were normalized to the most commonly used smallest package size (2"x3" for SURGICEL® Original and 1"x2" for advanced SURGICEL® products, respectively) to reconcile the discrepancy in the amount of ORC contained in different package sizes within each type of SURGICEL® product. Although 0.5"x2" was the smallest size for SURGICEL® Original, it was not selected as the standard package size because only 1 usage record was observed. Unit normalization as calculated as follows: normalized units = total surface area used per surgery / surface area of selected standard package size. Only patients with complete information of SURGICEL® size were included in this portion of the analysis. All SNoW® usages with missing size information were assigned to the size 2"x4", since only size 2"x4" was observed in SNoW® usage records.
2.6 Statistical analysis

SURGICEL® FIBRILLAR™ and SURGICEL® SNoW® were grouped as advanced SURGICEL® products due to their similar hemostasis time.[12] All measurements were compared between SURGICEL® Original and advanced SURGICEL® product cohorts. Continuous variables were compared between the two cohorts by fitting generalized linear models with Poisson, normal, negative binomial distribution with SURGICEL® hemostat product type as covariate. The Akaike information criterion (AIC) was used to choose the distribution assumption of each outcome, and the model with the lower value for AIC was selected as the final model. Categorical variables were compared between the two cohorts using Chi-squared test. The statistical significance was evaluated at 5% significance level.

Data management, analysis file preparation and descriptive statistical analyses were performed using SAS 9.3 (SAS Institute, Cary NC).

## RESULTS

### Cohort selection

A total of 2605 patients were identified from the EMR system of HFHS with keywords related to SURGICEL® products, of which 560 patients were excluded by applying each of the following exclusion criteria step by step: 1) received unidentifiable SURGICEL® product type (3 patients); 2) aged younger than 18 on index date (21 patients); 3) only received unrelated SURGICEL® products (489 patients); 4) received both SURGICEL® Original and Advanced SURGICEL® products (47 patients) (Figure 1). Of the remaining 2045 patients, 574 were further excluded due to unrelated surgeries and outpatient settings. Thus, 1,471 patients were included in the analysis.

**Figure 1:** Flowchart of study population selection

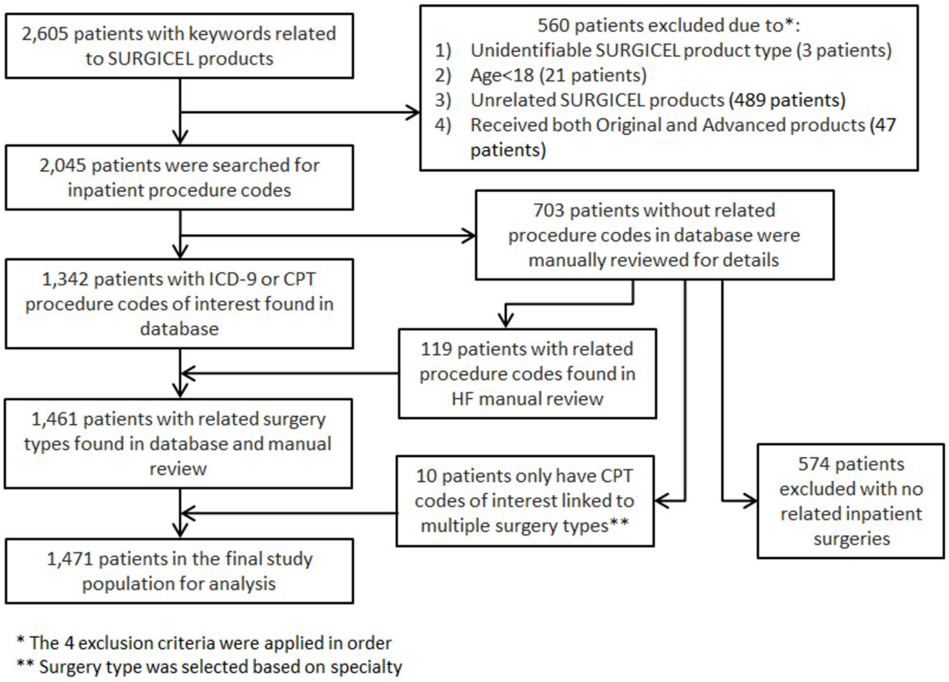

### Patient demographics and characteristics

Among 1,471 patients, 450 received SURGICEL® ORIGINAL (53.33% male), and 1,021 received advanced SURGICEL® products (either FIBRILLAR™ or SNoW®) (59.55% male) (Table 1). Advanced SURGICEL® products were observed to be used among more complex patients: a significantly greater proportion of patients given advanced SURGICEL® products had comorbidities (90.99% vs 85.56%, p=.0024), prior bleeding conditions (49.85% vs 30.89%, p<.0001), and prior use of anticoagulants (27.72% vs 5.33%, p<.0001) as compared to those who received SURGICEL® ORIGINAL (Table 1).

### Surgery type

The adoption of advanced SURGICEL® products was significantly different by surgery type (Table 2). Cardiovascular surgeons were the early adopters of advanced SURGICEL® products based on the large proportions of advanced SURGICEL® products used among cardiovascular surgeries compared to SURGICEL® Original (Coronary artery bypass grafting (CABG): 13.71% vs 1.56%, p<.0001; Valve replacement: 12.24% vs 0.67%, p<.0001; Valve replacement and CABG: 5.68% vs 0.22%, p<.0001). Advanced SURGICEL® products also were more likely to be used among laparoscopic cholecystectomy (14.10% vs 3.11%, p<.0001), nephrectomy (8.52% vs 3.33%, p=0.0002) and operations on kidney (9.99%vs 0.44%, p<.0001) compared to SURGICEL® Original. In contrast, advanced SURGICEL® products were less likely to be used during operations on thyroid and parathyroid glands (0.69% vs 7.56%, p<.0001), cerebral operations (1.96% vs 24.89%, p<.0001), laminectomy (0.49% vs 10.67% p<.0001) and lumbar fusions (including revisions) (0.20% vs 13.78%, p<.0001) compared to SURGICEL® Original.

**Table 1.** Patient demographics and characteristics, N=1471

| Demographics and characteristics | SURGICEL® Original n=450 | Advanced SURGICEL® n=1021 | P-value |
|---|---|---|---|
| **Age group, n (%)** | | | |
| 18 to <64 | 263 (58.44%) | 541 (52.99%) | 0.1525 |
| 64 to <80 | 149 (33.11%) | 384 (37.61%) | |
| ≥80 | 38 (8.44%) | 96 (9.40%) | |
| **Gender, n (%)** | | | |
| Male | 240 (53.33%) | 608 (59.55%) | 0.0295 |
| Female | 210 (46.67%) | 413 (40.45%) | |
| **Ethnicity, n (%)** | | | |
| Caucasian | 305 (67.78%) | 622 (60.92%) | 0.0196 |
| African American | 82 (18.22%) | 244 (23.90%) | |
| Hispanic | 6 (1.33%) | 29 (2.84%) | |
| Other/Unknown | 57 (12.67%) | 126 (12.34%) | |
| **Co-morbidities, n (%)** | | | |
| Any co-morbidities | 385 (85.56%) | 929 (90.99%) | 0.0024 |
| Myocardial infarction | 33 (7.33%) | 190 (18.61%) | <.0001 |
| Congestive heart failure | 41 (9.11%) | 244 (23.90%) | <.0001 |
| Peripheral vascular disease | 37 (8.22%) | 188 (18.41%) | <.0001 |
| Cerebrovascular disease | 100 (22.22%) | 136 (13.32%) | <.0001 |
| Dementia | 3 (0.67%) | 5 (0.49%) | 0.7064 |
| Chronic pulmonary disease | 93 (20.67%) | 284 (27.82%) | 0.0036 |
| Rheumatologic disease | 16 (3.56%) | 26 (2.55%) | 0.3088 |
| Peptic ulcer disease | 11 (2.44%) | 29 (2.84%) | 0.7311 |
| Liver disease | 8 (1.78%) | 47 (4.60%) | 0.0071 |
| Diabetes | 98 (21.78%) | 330 (32.32%) | <.0001 |
| Hemiplegia or paraplegia | 22 (4.89%) | 15 (1.47%) | 0.0004 |
| Renal disease | 97 (21.56%) | 364 (35.65%) | <.0001 |
| Any malignancy, including lymphoma and leukemia (including metastatic solid tumors) | 133 (29.56%) | 318 (31.15%) | 0.5808 |
| Metastatic solid tumor | 33 (7.33%) | 44 (4.31%) | 0.0216 |
| AIDS | 3 (0.67%) | 1 (0.10%) | 0.0880 |
| Obesity | 81 (18.00%) | 267 (26.15%) | 0.0007 |
| Hypertension | 268 (59.56%) | 749 (73.36%) | <.0001 |
| **Pre-existing bleeding condition, n (%)** | 139 (30.89%) | 509 (49.85%) | <.0001 |
| **Prior use of anticoagulants, n (%)** | 24 (5.33%) | 283 (27.72%) | <.0001 |

**Table 2.** Surgery type distribution, N=1471

| Distribution of surgery types, n (%) | SURGICEL® Original n=450 | Advanced SURGICEL® n=1021 | P-value |
|---|---|---|---|
| CABG[1] | 7 (1.56%) | 140 (13.71%) | <.0001 |
| Valve replacement | 3 (0.67%) | 125 (12.24%) | <.0001 |
| Valve replacement and CABG | 1 (0.22%) | 58 (5.68%) | <.0001 |
| Vascular shunt | 1 (0.22%) | 0 (0.00%) | 0.3059 |
| AV[2] graft/AV fistula | 0 (0.00%) | 2 (0.20%) | 1 |
| Fem-Pop bypass | 0 (0.00%) | 1 (0.10%) | 1 |
| CEA (Neck/head vessels-Carotid Endarterectomy) | 9 (2.00%) | 12 (1.18%) | 0.2192 |
| Lower extremity endarterectomy | 0 (0.00%) | 2 (0.20%) | 1 |
| CV[3] All others | 15 (3.33%) | 65 (6.37%) | 0.0176 |
| Vascular surgery, other | 9 (2.00%) | 31 (3.04%) | 0.3001 |
| Lobectomy of lung | 7 (1.56%) | 9 (0.88%) | 0.2781 |
| Operations on thyroid and parathyroid glands | 34 (7.56%) | 7 (0.69%) | <.0001 |
| Cerebral Operations | 112 (24.89%) | 20 (1.96%) | <.0001 |
| Laminectomy | 48 (10.67%) | 5 (0.49%) | <.0001 |
| Cholecystectomy, laparoscopic | 14 (3.11%) | 144 (14.10%) | <.0001 |
| Cholecystectomy, open | 9 (2.00%) | 26 (2.55%) | 0.5831 |
| Colectomy (Colorectal-right, transverse, sigmoid) w/ or w/out colostomy | 11 (2.44%) | 22 (2.15%) | 0.7060 |
| Small intestine resection | 2 (0.44%) | 5 (0.49%) | 1 |
| Adhesiolysis | 4 (0.89%) | 15 (1.47%) | 0.3638 |
| Nephrectomy | 15 (3.33%) | 87 (8.52%) | 0.0002 |
| Operations on kidney | 2 (0.44%) | 102 (9.99%) | <.0001 |
| Prostatectomy | 2 (0.44%) | 10 (0.98%) | 0.3643 |
| Hysterectomy | 2 (0.44%) | 11 (1.08%) | 0.3655 |
| Caesarean section | 4 (0.89%) | 14 (1.37%) | 0.4381 |
| Lumbar fusions (including revisions) | 62 (13.78%) | 2 (0.20%) | <.0001 |
| Orthopedic all others | 6 (1.33%) | 5 (0.49%) | 0.1020 |
| Multiple surgeries | 52 (11.56%) | 86 (8.42%) | 0.0650 |
| All others | 19 (4.22%) | 15 (1.47%) | 0.0021 |

[1]CABG: Coronary artery bypass grafting; [2]AV: Arteriovenous; [3]CV: Cardiovascular

### *Hospitalization characteristics*

Advanced SURGICEL® products were more likely to be used among severe surgical cases with longer mean length of hospital stay (10.71 vs 8.06 days, p<.0001) and longer mean length of ICU stay (10.00 vs 8.72 days, p=0.0220) compared to SURGICEL® Original (Table 3). The mean duration of surgery was longer among surgery cases that adopted advanced SURGICEL® products compared to SURGICEL® Original cases (238.14 vs 190.26 minutes, p<.0001). The surgery cases that experienced major bleeding events requiring more units of red blood and whole blood transfusions were more likely to use advanced SURGICEL® than SURGICEL® Original (red blood: 0.67 vs 0.30 units, p=<.0001; whole blood: 0.11 vs 0.02 units, p=<.0001). Advanced SURGICEL® products were more likely to be used in severe surgical cases requiring a blood transfusion on surgery day than SURGICEL® Original (24.29% vs 14.00%, p<.0001).

**Table 3.** Surgery case characteristics by product type, N=1471

| | LSMEAN (95% CI)[1] | | |
|---|---|---|---|
| **Hospitalization characteristics** | **SURGICEL® Original n=450** | **Advanced SURGICEL® n=1021** | **p-value** |
| Length of hospital stay (days) | 8.06 [7.35,8.84] | 10.71 [10.08,11.37] | <.0001 |
| Length of ICU stay (days) | 8.72 [7.91,9.62] | 10.00 [9.39,10.65] | 0.0220 |
| Duration of surgery (minutes) | 190.26 [179.38,201.80] | 238.14 [229.25,247.39] | <.0001 |
| Units of red blood transfused per surgery | 0.30 [0.21,0.42] | 0.67 [0.54,0.83] | <.0001 |
| Units of whole blood transfused per surgery | 0.02 [0.01,0.04] | 0.11 [0.09,0.13] | <.0001 |
| | **Categorical variables, n (%)** | | |
| **Hospitalization outcomes** | **SURGICEL® Original n=450** | **Advanced SURGICEL® n=1021** | **p-value** |
| Blood transfusion on surgery day | 63 (14.00%) | 248 (24.29%) | <.0001 |
| Procedures to control bleeding on surgery day | 10 (2.22%) | 37 (3.62%) | 0.1980 |
| Procedures to control bleeding after surgery day | 11 (2.44%) | 38 (3.72%) | 0.2693 |
| Post-surgery death | 15 (3.33%) | 44 (4.31%) | 0.4710 |

[1]LSMEAN was the least squares mean derived from the generalized linear model, and the sample mean was directly calculated from the study population.

### Normalized units

Among a sub-set of 1,420 patients with complete SURGICEL® package size information (including 988 advanced SURGICEL® and 432 SURGICEL® Original), significantly fewer mean normalized units of advanced SURGICEL® products were used per patient case as compared to SURGICEL® Original (3.86 vs 5.49 units, p<.0001). The adjusted analysis of normalized units with demographics and surgery type as covariates had similar result to the unadjusted analysis.

**Figure 2.** Unadjusted analysis of normalized units of SURGICEL® used per surgery1, N=1420

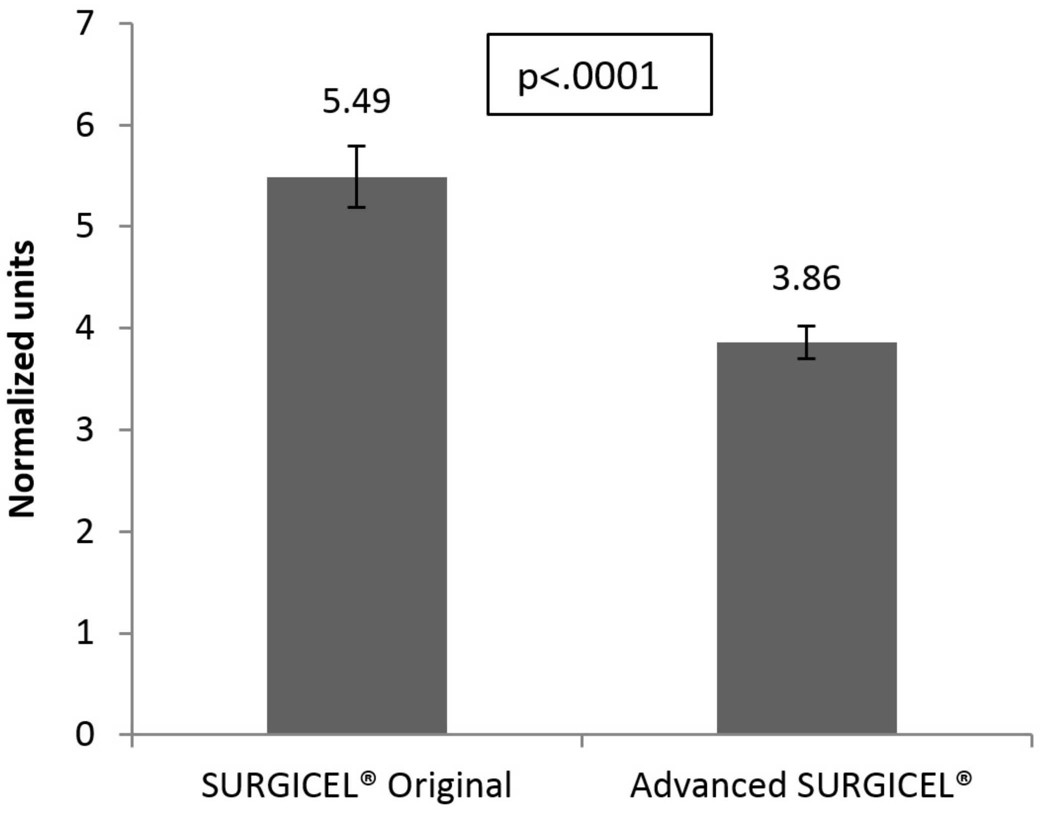

[1]Units were normalized to package 2”x3” for Original and package 1”x2” for Advanced respectively.
Equation for unit normalization: Normalized units=Total surface area used per surgery / surface area of standard package size. On average, 3.86 units of Advanced SURGICEL® (1”x2”) were used for each surgery, while 5.49 units (2”x3”) of SURGICEL® Original were used per surgery (p<.0001). Only patients with complete package size information were included in the analysis.

## DISCUSSION

We described the real-world utilization pattern of increased adoption of advanced SURGICEL® products in comparison with SURGICEL® Original in a vertically integrated healthcare system using data collected from EMRs. In this study, we found that advanced SURGICEL® products were more likely to be used among patients with comorbidities, prior bleeding conditions, and prior use of anticoagulants. This suggested that surgeons observed here had a tendency to use advanced SURGICEL® products for patients with higher risk profiles possibly in order to achieve better management of bleeding. Advanced SURGICEL® products were also more likely to be adopted among surgery cases with higher resource utilization, including length of hospital stay, ICU stay, duration of surgery, and units of red blood cell and whole blood transfusions. These results were aligned with patient risk profiles within each treatment cohort, which altogether imply that advanced SURGICEL® products are more likely to be used in more complex surgical cases and among higher risk patients.

Adoption trends, in this hospital, of advanced SURGICEL® products varied by surgery type and surgical specialty. Cardiovascular surgeons appeared to be early adopters of advanced SURGICEL® products. Such phenomena may potentially be driven by the imperative demand for advanced hemostatic products with high efficiency among patients at higher risk for bleeding. Similar preference to advanced SURGICEL® products was also observed among laparoscopic cholecystectomy, nephrectomy and operations on kidney. However, SURGICEL® Original was still used as the main topical hemostatic product in operations on thyroid and parathyroid glands, cerebral operations, laminectomy and lumbar fusions (including revisions). To better cope with the problem of surgical bleeding, the use of appropriate hemostatic products with the right procedure requires further research and continuing education.

Although advanced SURGICEL® products were mostly used in surgical cases with higher bleeding risk, fewer overall normalized units of advanced SURGICEL® products were used in each surgery compared to SURGICEL® Original, which implies that surgeons are making informed decisions on the appropriate use of hemostats taking into account bleeding risk as well as resource efficiency.

Since advanced SURGICEL® products were largely used in more complex surgical cases and in higher risk patients, it was difficult to assess the advanced SURGICEL® products' impact on health outcomes and resource utilization. Further research is needed to investigate the implications of the efficiency of topical adjunctive hemostats on patient outcomes, hospital costs, and resource utilization in continuous oozing bleeding situations as this study was not designed to make any causal conclusions.

## CONCLUSIONS

In conclusion, advanced SURGICEL® products were observed as more likely to be used in patients with higher risk profiles, including comorbidities, prior bleeding conditions, and prior use of anticoagulants. Furthermore, advanced SURGICEL® products were more highly adopted in cardiovascular surgeries and more complex surgical cases. Despite advanced SURGICEL® products being utilized in higher risk bleeding situations, fewer overall normalized units were required per patient case as compared to SURGICEL® ORIGINAL, suggesting the utility of these topical adjunctive hemostats. These observations warrant further investigation into the implications of topical hemostat efficiency on health outcomes, hospital resource utilization, and hospital costs.

## DISCLOSURES

### Declaration of funding

Funding for this study was provided by Ethicon, Inc. GG, RK and WD are employees of Ethicon, Inc.
